# Antibacterial Activity and Cytotoxic Effect of *Pelargonium peltatum* (Geranium) against *Streptococcus mutans* and *Streptococcus sanguinis*

**DOI:** 10.1155/2018/2714350

**Published:** 2018-11-28

**Authors:** Samantha Coronado-López, Stefany Caballero-García, Miguel Angel Aguilar-Luis, Fernando Mazulis, Juana del Valle-Mendoza

**Affiliations:** ^1^School of Dentistry, Faculty of Health Sciences, Universidad Peruana de Ciencias Aplicadas, Lima, Peru; ^2^School of Medicine, Research Center and Innovation of the Faculty of Health Sciences, Universidad Peruana de Ciencias Aplicadas, Lima, Peru; ^3^Laboratorio de Biología Molecular, Instituto de Investigación Nutricional, Lima, Peru

## Abstract

**Objective:**

To evaluate the *in vitro* antibacterial and cytotoxic properties of the methanolic extract of *Pelargonium peltatum* (geranium) against *Streptococcus mutans* (ATCC 25175) and *Streptococcus sanguinis* (ATCC 10556).

**Methods:**

Three extracts of *P. peltatum* were prepared using the leaf, stem, and root. Nine independent assays were prepared for each type of extract with chlorhexidine at 0.12% as the positive control. The agar diffusion method was performed to determine the antibacterial properties of each extract. The minimum inhibitory concentration (MIC) was determined using the microdilution method, and the cytotoxicity was analyzed by means of the MTT reduction test using a MDCK cell line.

**Results:**

The root extract had the highest antibacterial effect with a mean result of (27.68 ± 0.97) mm and (30.80 ± 0.55) mm against *S. mutans* and *S. sanguinis*, respectively. The minimum inhibitory concentration for the leaf and root extracts was 250 mg/mL for *S. mutans* and 125 mg/mL for *S. sanguinis*. Cytotoxicity assays showed that both extracts had a low cytotoxicity at high concentrations. The cellular viability was highest for the root extract at 95.3% followed by the stem extract at 80.8% and finally the leaf extract with 75.4%.

**Conclusions:**

These findings show the antibacterial properties of the methanolic extracts of *P. pelargonium* against *S. mutans* and *S. sanguinis*. These extracts were not cytotoxic at high concentrations.

## 1. Introduction

Prioritization on prevention and promotion of health in dentistry, as well as for many other medical specialties, is of great importance to reduce the risk of infection of the most prevalent diseases [1–4]. In Peru, dental caries is a major oral health problem with a high prevalence in the population [5, 6].

Recent scientific breakthroughs have been able to determine that the main factor associated to this disease is the demineralization caused by the acidic by-products of oral fermenting bacteria. Therefore, acidophilic bacteria such as *Streptococcus mutans*, *Streptococcus sanguinis*, and *Lactobacillus* are required for dental decay to occur [7–12]. Various treatment options have been developed and are currently used in dentistry as first-line therapies for this condition. These include topical fluorine, fluoride varnish, toothpaste, and mouthwashes [2, 3].

Despite current means to diagnose and treat this condition, the prevalence of dental caries in Peru remains high. It is therefore necessary to develop new treatment alternatives that may complement current therapies. Phytotherapy research focuses on studying the medicinal properties offered by plants and vegetables substances, to treat and improve treatment outcomes of certain diseases [13–15].

*Pelargonium peltatum*, commonly known as ivy geranium, is a plant that was recently introduced to the field of dentistry. Even though its properties have been studied and applied in the field of medicine for the treatment of respiratory diseases such as bronchitis and sinusitis, few studies have evaluated its effectiveness against bacteria responsible for diseases of the oral cavity [16–18].

Given the high prevalence of dental caries in Peru, it is important to consider phytotherapy as a treatment alternative in the field of odontology. The purpose of this study was to evaluate the antibacterial and cytotoxic effects of the methanolic extracts of *P. peltatum* against *S. mutans* and *S. sanguinis*.

## 2. Materials and Methods

### 2.1. Extract Preparation

*P. peltatum* (ivy geranium) was acquired from the university herb garden of Universidad Nacional Agraria La Molina (UNALM) in the city of Lima, Peru. Three different extracts were prepared using the roots, stems, and leaves of the studied plant. These products were separated in different containers and immersed independently in absolute methanol (1 : 1 weight/volume). The samples were then stored at room temperature for 7 days without sunlight exposure. The 3 different extract solutions were filtered through Whatman® qualitative filter paper, grade 4 (20–25 *µ*m), and placed in labeled tubes. The tubes were distilled using a rotatory evaporator at 50°C to obtain a pure extract. The extracts were stored in 15 mL plastic tubes in a rack without sunlight exposure and at a temperature of 4°C until further use.

### 2.2. Bacterial Cultures

The anaerobic standard strains employed in the study were provided by the American Type Culture Collection: *S. mutans* (ATCC 25175) and *S. sanguinis* (ATCC 10556). These were identified by species and genre.

The bacteria were cultured on BHI (brain heart infusion) agar plates and incubated at 37°C for 72 hours in anaerobic conditions. We proceeded to isolate 3 to 4 colonies of which were later inoculated in a 3 mL BHI broth. The cultures were diluted in a sterile saline solution to a McFarland scale density of 0.5, which corresponds to a concentration of approximately 1.5 × 10^8^ UFC/mL [19].

### 2.3. Antibacterial Activity of Methanolic Extracts

The antibacterial effect was analyzed by means of the well diffusion method. We autoclaved the BHI agar medium plates at 121°C for 15 minutes. After cooling, the bacterial suspension was inoculated on the agar plates. A sterile cork borer was used to punch 8 mm-diameter wells which were then filled with 100 *µ*L of each extract. We used chlorhexidine 0.12% [20] as a positive control for each assay. The whole procedure was done in a laminar flow cabin in order to maintain sterile conditions. The agar plates were incubated in anaerobic conditions at 37°C for 48 days.

### 2.4. Determination of the Minimum Inhibitory Concentration (MIC)

The minimum inhibitory concentration of each extract was determined using the microdilution method following the recommendations by the Clinical Laboratory Standard Institute (CSLI, 2012) [21]. Each methanolic extract was diluted to concentrations ranging between 0 mg/mL to 500 mg/mL and then added separately to the wells. Then, 100 *µ*L of each bacterial suspension (0.5 McFarland scale) was added to these wells and then incubated at 37°C for 48 hours in anaerobic conditions. The minimum inhibitory concentration was defined as the minimum concentration of the extract required to inhibit bacterial growth.

### 2.5. Cytotoxicity Evaluation of *P. peltatum*

#### 2.5.1. Cell Line Cultures

The MDCK cell lines were obtained from ATCC (American Type Culture Collection). The cells were culture in an essential culture medium with Earle's salts (Gibco BRL, Grand Island, NY), enriched with fetal bovine serum at 10%, 25 *µ*g/L of gentamicin, and 200 mmol/L of L-glutamine (growth medium). The infected cells were stored in a minimum essential medium with fetal bovine serum at 1%, 25 *µ*g/L of gentamicin, and 200 mmol/L of L-glutamine (maintenance medium). All the cells were cultured at 37°C in a humidified atmosphere with 5% of CO_2_ and 95% air.

#### 2.5.2. Cytotoxicity

An MTT reduction assay was used to determine the cytotoxicity of the extracts on MDCK cell lines. The cell lines were cultured on a sterile 96-well microtiter plate. Subsequently, we proceeded to distribute varying concentrations of each methanolic extract of *P. peltatum* onto the plate. The concentration of each extract ranged between 5 *µ*g/mL to 100 *µ*g/mL. A culture medium without any extract was used as the positive control for this cellular viability. The plate was incubated at 37°C for 6 days. The cellular viability of each extract was determined as a percentage of the absorbance obtained for each well in comparison with the positive control. The absorbance values (570 mm) were measured using a photometric reader.

## 3. Results

### 3.1. Antibacterial Effect of *P. peltatum* Extracts

The results obtained from this study showed that the extracts had a significant antibacterial property.  Table 1 shows that the root extract of *P. peltatum* had the highest antibacterial effect against *S. mutans* with an inhibitory zone of 27.68 mm followed by the leaf and the stem extracts with inhibitory zones of 25.85 mm and 23.97 mm, respectively.

On the other hand, the root extract had the highest antibacterial effect against *S. sanguinis* with a mean inhibitory zone of 30.8 mm followed by the leaf extracts with a mean value of 29.6 mm and finally the stem extract with 27.54 mm. We found statistically significant differences using the Kruskal–Wallis test (*p* < 0.01).

### 3.2. Minimum Inhibitory Concentration

The minimum inhibitory concentration (MIC) of the methanolic extracts against *S. mutans* strains was 250 mg/mL for the leaf and root extracts and 125 mg/mL for the stem extracts. In contrast, the MIC of the root and leaf extracts against *S. sanguinis* was 125 mg/mL and 31 mg/mL for the stem extract (Table 2).

### 3.3. Cytotoxicity of the *P. peltatum* Extract

**Figure 1:**
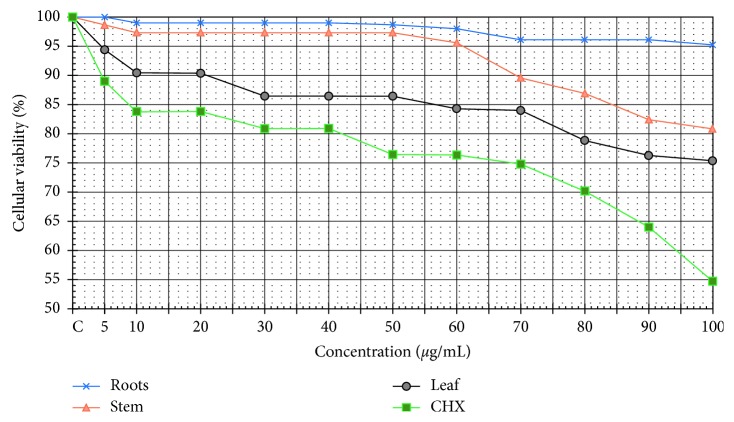
Cellular viability evaluation of the methanolic extracts of *Pelargonium peltatum* using MCDK cell line.

The results indicate that the methanolic extracts of *P. peltatum* inhibit 50% of cellular viability (CC_50_) at a higher concentration of 100 *µ*g/ml. Furthermore, none of the extracts produced any adverse effects on the MDCK cellular lines at low concentrations. We observed an indirect correlation between the concentration and cellular viability (Figure 1).

## 4. Discussion

Medicine has evolved over the years providing the world with new therapies that contribute to the well-being of the population. Phytotherapy, the use of natural products as an alternative to treat different ailments, is being continually developed [13].

*S. mutans* and *S. sanguinis* are commonly parts of bacterial flora in the oral cavity. These microorganisms are required in the pathogenesis of certain prevalent diseases, such as dental caries [22]. Furthermore, these bacteria are also associated with periodontal disease and systemic disease such as bacteremia and endocarditis, as is the case for *S. sanguinis* [23].

In this study, we showed that the plant *P*. *peltatum* has high antibacterial properties against microorganisms responsible for prevalent diseases of the oral cavity. The results coincide with the data obtained by similar studies, such as Guerrero Hurtado et al. [24], in which the antibacterial effect of an aqueous extract of a geranium plant was evaluated against the strains of *S. mutans*, *S. sanguinis*, and *Streptococcus mitis* showing antibacterial effect of the extracts against all the studied strains. The antibacterial effect of *Pelargonium peltatum* can be explained by the antimicrobial activity of its phytoconstituents, which include flavonoids, tannins, steroids, anthocyanins, quinones, and saponins. Tannins were found in greater concentration in the roots and cortex of these plants and have shown to have effective antiseptic, antibacterial, and astringent properties [18, 25]. This could explain the higher antibacterial effect observed with root extracts.

It is worth noting that there are many species of the genus *Pelargonium*; other studies have analyzed the antibacterial activity of the plants belonging to this family but very few to the species used in this study. Ghannadi et al. [16] evaluated the antibacterial effect of two plants, one of them being *Pelargonium graveolens*, against 6 microorganisms and showed that 5 of them were susceptible to the oil extract of this geranium class.

Galea and Hancu [17] conducted a similar research showing the antibacterial effect of *Pelargonium roseum* against 6 strains of Gram-negative bacteria and the fungus *Candida albicans*, a dimorphic fungus included in the natural oral flora that causes disease in immunosuppressed patients.

The minimum inhibitory concentration (MIC) found in this study was found to be between 125 mg/mL to 250 mg/mL. We could not find studies that found the MIC of geranium against the microorganisms in this study. However, in a study conducted by Bigos et al. [18] in which geranium antibacterial effect was evaluated on strains of *Staphylococcus aureus*, it was observed that the MIC was 250 mg/mL, data similar to those obtained in this study.

We evaluated the cytotoxicity of the extracts against the cell line MDCK and observed promising results. The cytotoxicity assay showed a cell viability of 95.3%, 74.4%, and 80.4% for the root, stem, and leaf extracts, respectively, at high concentrations (100 *µ*g/mL). We could not find previously published studies on the cytotoxicity of these extracts.

Geranium is a plant that is easy to grow and is economically and logistically accessible [17] and that possess high antibacterial properties with low cytotoxicity at high concentrations. The use of this plant could be considered as a new alternative in the development of effective toothpastes or mouthwashes. Further experimental and clinical studies are required to guarantee its safety and to further evaluate the antibacterial properties *in vivo*.

The results in this study have demonstrated that the geranium plant has antibacterial properties associated with a low cytotoxicity, and furthermore, it has a low production cost and is readily accessible. These properties make the extracts of geranium promising candidates for the development of novel dental products.

## 5. Conclusions

The findings in this study show that the methanolic extracts of *P. peltatum* (geranium) has effective antibacterial properties against strains of *S. mutans* and *S. sanguinis*. Additionally, we found that the root extracts had the greatest antibacterial effect in comparison with the leaf and stem extracts. The cytotoxicity assay showed a high cellular viability at high concentrations. We suggest further studies with this species of *Pelargonium* given that research is lacking on the matter.

## Figures and Tables

**Table 1:** *In vitro* comparison of the antibacterial effect of the methanolic extracts of *Pelargonium peltatum* against strains of *Streptococcus mutans* and *Streptococcus sanguinis.*

Microorganism	Extract	Inhibition zone (mm)				
		Mean	SD	Minimum	Maximum	*p* value^*∗*^
*Streptococcus mutans*	Root	27.68	0.97	25.5	28.8	<0.01
	Leaf	25.86	0.54	24.6	26.3	
	Stem	23.97	0.91	22.5	25	

*Streptococcus sanguinis*	Root	30.8	0.55	30	32.1	<0.01
	Leaf	29.26	0.76	27.8	30.5	
	Stem	27.53	1.13	26	29.4	

^*∗*^Kruskal–Wallis test.

**Table 2:** Minimum inhibitory concentration according to the methanolic extract of *Pelargonium peltatum* against strains of *Streptococcus mutans* (ATCC 25175) and *Streptococcus sanguinis* (ATCC 10556).

Microorganism	Extract	Concentration (mg/mL)									
		500	250	125	62.5	31	16	8	4	2	1
*Streptococcus mutans*	Leaf	+	MIC	−	−	−	−	−	−	−	−
	Stem	+	+	MIC	−	−	−	−	−	−	−
	Roots	+	MIC	−	−	−	−	−	−	−	−
	CHX	+	+	+	+	+	+	+	+	MIC	−

*Streptococcus sanguinis*	Leaf	+	+	MIC	−	−	−	−	−	−	−
	Stem	+	+	+	+	MIC	−	−	−	−	−
	Roots	+	+	MIC	−	−	−	−	−	−	−
	CHX	+	+	+	+	+	+	MIC	−	−	−

^*∗*^2CHX: chlorhexidine, MIC: minimum inhibitory concentration, (**+**): extract inhibit the visible growth of strains, (−): extract not inhibit the visible growth of strains.

## Data Availability

Abstraction format used in the study and dataset are available and accessible from the corresponding author upon request.

## References

[1] Petersen P. E. (2003). *The World Oral Health Report 2003. Continuous Improvement of Oral Health in the 21st Century–the Approach of the WHO Global Oral Health Programme*.

[2] Tarvonen P. L., Sipilä K., Yang G. S. (2016). Comparison of two preventive interventions on dental caries among children in democratic people’s Republic of Korea. *International Journal of Dental Hygiene*.

[3] de Silva A. M., Hegde S., Akudo Nwagbara B. (2016). Community-based population-level interventions for promoting child oral health. *Cochrane Database of Systematic Reviews*.

[4] Huebner C. E., Milgrom P. (2015). Evaluation of a parent-designed programme to support tooth brushing of infants and young children. *International Journal of Dental Hygiene*.

[5] Pulache J., Abanto J., Oliveira L. B. (2016). Exploring the association between oral health problems and oral health-related quality of life in Peruvian 11 to 14-year-old children. *International Journal of Paediatric Dentistry*.

[6] Delgado-Angulo E. K., Hobdell M. H., Bernabé E. (2013). Childhood stunting and caries increment in permanent teeth: a three and a half year longitudinal study in Peru. *International Journal of Paediatric Dentistry*.

[7] Garcia S. S., Du Q., Wu H. (2016). *Streptococcus mutans* copper chaperone, CopZ, is critical for biofilm formation and competitiveness. *Molecular Oral Microbiology*.

[8] Merritt J J., Qi F. (2012). The mutacins of *Streptococcus mutans*: regulation and ecology. *Molecular Oral Microbiology*.

[9] Misaki T., Naka S., Hatakeyama R. (2016). Presence of *Streptococcus mutans* strains harbouring the CNM gene correlates with dental caries status and IgA nephropathy conditions. *Scientific Reports*.

[10] Kreth J., Giacaman R. A., Raghavan R. (2017). The road less traveled–defining molecular commensalism with *Streptococcus sanguinis*. *Molecular Oral Microbiology*.

[11] Mitrakul K., Vongsawan K., Sriutai A. (2016). Association between *S. mutans* and *S. sanguinis* in severe early childhood caries and caries-free children a quantitative real-time PCR analysis. *Journal of Clinical Pediatric Dentistry*.

[12] Mitrakul K., Asvanund Y., Vongsavan K. (2011). Prevalence of five biofilm-related oral streptococci species from plaque. *Journal of Clinical Pediatric Dentistry*.

[13] Yadav R., Rai R., Yadav A. (2016). Evaluation of antibacterial activity of *Achyranthes aspera* extract against *Streptococcus mutans*: an in vitro study. *Journal of Advanced Pharmaceutical Technology and Research*.

[14] Medina-Flores D., Ulloa-Urizar G., Camere-Colarossi R. (2016). Antibacterial activity of *Bixa orellana L.* (achiote) against *Streptococcus mutans* and *Streptococcus sanguinis*. *Asian Pacific Journal of Tropical Biomedicine*.

[15] Camere-Colarossi R., Ulloa-Urizar G., Medina-Flores D. (2016). Antibacterial activity of *Myrciaria dubia* (*Camu camu*) against *Streptococcus mutans* and *Streptococcus sanguinis*. *Asian Pacific Journal of Tropical Biomedicine*.

[16] Ghannadi A., Bagherinejad M. R., Abedi D. (2012). Antibacterial activity and composition of essential oils from *Pelargonium graveolens* L’Her and *Vitex agnus-castus* L. *Iranian Journal of Microbiology*.

[17] Carmen G., Hancu G. (2014). Antimicrobial and antifungal activity of *Pelargonium roseum* essential oils. *Advanced Pharmaceutical Bulletin*.

[18] Bigos M., Wasiela M., Kalemba D. (2012). Antimicrobial activity of *geranium* oil against clinical strains of *Staphylococcus aureus*. *Molecules*.

[19] Andrews J. M., Howe R. A. (2011). BSAC working party on susceptibility testing. BSAC standardized disc susceptibility testing method (version 10). *Journal of Antimicrobial Chemotherapy*.

[20] Karuppiah P., Rajaram S. (2012). Antibacterial effect of *Allium sativum* cloves and *Zingiber officinale* rhizomes against multiple-drug resistant clinical pathogens. *Asian Pacific Journal of Tropical Biomedicine*.

[21] CLSI (2012). *Methods for Dilution Antimicrobial Susceptibility Tests for Bacteria that Grow Aerobically*.

[22] Kouidhi B., Al Qurashi Y. M., Chaieb K. (2015). Drug resistance of bacterial dental biofilm and the potential use of natural compounds as alternative for prevention and treatment. *Microbial Pathogenesis*.

[23] Babii C., Bahrin L. G., Neagu A. N. (2016). Antibacterial activity and proposed action mechanism of a new class of synthetic tricyclic flavonoids. *Journal of Applied Microbiology*.

[24] Guerrero Hurtado J., Ortiz Rubio Z. M., Peralta Berrospi L. F. (2013). Actividad antibacteriana del *Pelargonium peltatum* L’Her. Sobre *Streptococcus mutans, Streptococcus sanguinis* y *Streptococcus mitis* frente a la clorhexidina. *Revista Cubana de Plantas Medicinales*.

[25] Lis-Balchin M. (2003). *Geranium and Pelargonium: History of Nomenclature, Usage and Cultivation*.

